# Supplementary material for: Resolving the full spectrum of human genome variation using Linked-Reads
Source: Genome Res. 2019 Apr;29(4):635–45. doi: 10.1101/gr.234443.118 (PMC6442396; doi:10.1101/gr.234443.118)
Supplement: Supplemental Material [file supp_gr.234443.118_Supplemental_longranger-2.2.zip › longranger-2.2.tar.bz2!/lr2.2-public/lib/stan/cmdstan/stan/doc/api/html/ast__def_8cpp.html]

Stan: src/stan/gm/ast\_def.cpp File Reference


|  |  |
| --- | --- |
|  | Stan  2.5.0  probability, sampling & optimization |


- Main Page
- Related Pages
- Namespaces
- Classes
- Files

- File List
- File Members

All Classes Namespaces Files Functions Variables Typedefs Enumerations Enumerator Friends Macros Pages

- src
- stan
- gm

Namespaces |
Macros |
Functions

ast\_def.cpp File Reference

`#include <boost/variant/apply_visitor.hpp>`  
`#include <boost/variant/recursive_variant.hpp>`  
`#include <cstddef>`  
`#include <limits>`  
`#include <climits>`  
`#include <iostream>`  
`#include <map>`  
`#include <stdexcept>`  
`#include <string>`  
`#include <utility>`  
`#include <vector>`  
`#include <stan/gm/ast.hpp>`  
`#include <stan/gm/function_signatures.h>`

Go to the source code of this file.

|  |  |
| --- | --- |
| Namespaces | |
|  | stan |
|  | Probability, optimization and sampling library. |
|  | |
|  | stan::gm |
|  | Directed graphical model compiler. |
|  | |

|  |  |
| --- | --- |
| Macros | |
| #define | \_\_STAN\_\_GM\_\_AST\_DEF\_HPP\_\_ |
|  | |

|  |  |
| --- | --- |
| Functions | |
| std::ostream & | stan::gm::write\_base\_expr\_type (std::ostream &o, base\_expr\_type type) |
|  | |
| std::ostream & | stan::gm::operator<< (std::ostream &o, const expr\_type &et) |
|  | |
| expr\_type | stan::gm::promote\_primitive (const expr\_type &et) |
|  | |
| expr\_type | stan::gm::promote\_primitive (const expr\_type &et1, const expr\_type &et2) |
|  | |
| bool | stan::gm::returns\_type (const expr\_type &return\_type, const statement &statement, std::ostream &error\_msgs) |
|  | |
| bool | stan::gm::is\_linear\_function (const std::string &name) |
|  | |
| bool | stan::gm::has\_var (const expression &e, const variable\_map &var\_map) |
|  | |
| bool | stan::gm::has\_non\_param\_var (const expression &e, const variable\_map &var\_map) |
|  | |
| bool | stan::gm::is\_nil (const expression &e) |
|  | |
| size\_t | stan::gm::total\_dims (const std::vector< std::vector< expression > > &dimss) |
|  | |
| expr\_type | stan::gm::infer\_type\_indexing (const base\_expr\_type &expr\_base\_type, size\_t num\_expr\_dims, size\_t num\_index\_dims) |
|  | |
| expr\_type | stan::gm::infer\_type\_indexing (const expression &expr, size\_t num\_index\_dims) |
|  | |
| void | stan::gm::print\_var\_origin (std::ostream &o, const var\_origin &vo) |
|  | |
| bool | stan::gm::has\_rng\_suffix (const std::string &s) |
|  | |
| bool | stan::gm::has\_lp\_suffix (const std::string &s) |
|  | |
| bool | stan::gm::is\_user\_defined (const std::string &name, const std::vector< expression > &args) |
|  | |
| bool | stan::gm::is\_user\_defined\_prob\_function (const std::string &name, const expression &variate, const std::vector< expression > &params) |
|  | |
| bool | stan::gm::is\_user\_defined (const fun &fx) |
|  | |
| bool | stan::gm::is\_assignable (const expr\_type &l\_type, const expr\_type &r\_type, const std::string &failure\_message, std::ostream &error\_msgs) |
|  | |
| bool | stan::gm::ends\_with (const std::string &suffix, const std::string &s) |
|  | |

## Macro Definition Documentation

#define \_\_STAN\_\_GM\_\_AST\_DEF\_HPP\_\_

Definition at line 2 of file ast\_def.cpp.


---

[ Stan Home Page ]

*© 2011–2014,
Stan Development Team.*
